# Mediterranean diet in the southern Croatia – does it still exist?

**DOI:** 10.3325/cmj.2016.57.415

**Published:** 2016-10

**Authors:** Ivana Kolčić, Ajka Relja, Andrea Gelemanović, Ana Miljković, Kristina Boban, Caroline Hayward, Igor Rudan, Ozren Polašek

**Affiliations:** 1 University of Split, School of Medicine, Split, Croatia; 2 Medical student, University of Split School of Medicine, Split, Croatia; 3 Institute for Genetics and Molecular Medicine, University of Edinburgh, Edinburgh, UK; 4 Centre for Global Health Research, Usher Institute, University of Edinburgh, Edinburgh, UK; * The first two authors contributed equally.

## Abstract

**Aim:**

To assess the adherence to the Mediterranean diet in the population of Dalmatia in southern Croatia.

**Methods:**

A cross-sectional study was performed within the 10 001 Dalmatians cohort, encompassing 2768 participants from Korčula and Vis islands and the City of Split, who were recruited during 2011-2014. Using the data obtained from food frequency questionnaire we calculated the Mediterranean Diet Serving Score (MDSS). Multivariate logistic regression was used to identify the characteristics associated with the adherence to the Mediterranean diet, with age, sex, place of residence, education attainment, smoking, and physical activity as covariates.

**Results:**

The median MDSS score was 11 out of 24 points (interquartile range 8-13), with the highest score recorded on the island of Vis. Participants reported a dietary pattern that had high compliance with the Mediterranean diet guidelines for consumption of cereals (87% met the criteria), potatoes (73%), olive oil (69%), and fish (61%), moderate for consumption of fruit (54%) and vegetables (31%), and low for consumption of nuts (6%). Overall, only 23% of the participants were classified as being adherent to the Mediterranean diet, with a particularly low percentage among younger participants (12%) compared to the older ones (34%). Men were less likely to show good adherence (odds ratio 0.52, 95% confidence interval 0.42-0.65).

**Conclusion:**

This study revealed rather poor compliance with the current recommendations on the Mediterranean diet composition in the population of Dalmatia. Public health intervention is especially needed in younger age groups and in men, who show the greatest departure from traditional Mediterranean diet and lifestyle.

Mediterranean diet is one of the most commonly investigated nutritional patterns, marked by numerous beneficial health effects. It is traditionally practiced in countries of the Mediterranean basin, especially Greece, Italy, and Spain (1). Described benefits included cardiovascular diseases prevention (2), reversion of the metabolic syndrome (3), prevention of the invasive breast cancer (4), prostate (5), and colorectal cancer (6), prevention of the age-related cognitive decline (7), and even a protective role in asthma among children (8,9). Additionally, in Swedish population a 2-year survival increase was observed among people with higher compliance with the Mediterranean diet (10). Mediterranean diet was also shown to reduce the risk of cardiovascular and all-cause mortality both in people diagnosed with diabetes type 2 (11) and in healthy population (12). However, a Cochrane systematic review that included randomized controlled trials published until 2012 found only a modest effect of the Mediterranean diet or its components on the cardiovascular risk factors reduction (13), possibly due to methodological differences and design limitations of the published studies. More recently, results from the PREDIMED study, a large randomized controlled field trial in individuals at high risk of cardiovascular disease became available (14), confirming the beneficial effects of the Mediterranean diet on cardiometabolic health (15) and its role in the primary prevention of cardiovascular diseases (16).

The dietary pattern and compliance with the Mediterranean diet has so far been assessed using many definitions and various scoring systems (17). This is a consequence of several factors, including the availability and types of locally produced foods, lifestyle, and tradition (18). The most prominent characteristics of the Mediterranean diet are the use of olive oil (preferably virgin oil) as the primary fat source, abundant consumption of vegetables, fruits, nuts, and whole grains daily, with moderate red wine and legumes consumption. Animal products are more of a relish, and the priority is given to fish and white meat over red and processed meat (19). Recently, a new scoring system has been proposed to assess the individual compliance with the Mediterranean diet – Mediterranean Diet Serving Score (MDSS), claimed to be easy, valid, and accurate instrument to assess the Mediterranean diet adherence based on the consumption of foods and food groups per meal, day, and week (20). Its advantage is that it includes as many as 14 groups of foods, adding 1, 2, or 3 points to the total score based on the consumption frequency and the relative importance of the particular foods, without assigning negative points (20).

Nutritional transition, marked with the rapid spread of highly processed foods rich in sugar and saturated fats and fast food, as opposed to the home cooking (21), coupled with the sedentary lifestyle, is believed to be the driving force behind the pandemic of chronic diseases like obesity, diabetes, and cardiovascular diseases. Unfortunately, these trends have also spread to the Mediterranean countries, resulting in a switch from the traditional diet toward western diets (22).

Mediterranean diet and its particular composition in the Dalmatian population has previously been marginally investigated, using various study approaches and scoring (23,24). The aim of this study was to assess the compliance with the Mediterranean dietary pattern and its constituting components in the population of the island of Korčula, island of Vis, and the City of Split, Croatia. This is the first study that uses a systematic and validated approach to estimate the prevalence and factors associated with the adherence to the Mediterranean diet in a large population-based sample, and thus provide a reliable source of information for international comparisons and monitoring of nutritional trends and transition.

## Materials and methods

This cross-sectional study was performed within the “10,001 Dalmatians” cohort study (25,26). “10,001 Dalmatians” study was initiated in 1999, and has been investigating the health of isolated island communities ever since (27). This descriptive cross-sectional study included only participants recruited after 2010 during either their follow-up or upon their first enrolment, in order to portrait the contemporary dietary patterns. The participants originated from the island of Vis (N = 401; recruited in 2011), the island of Korčula (N = 1980; recruited in 2012-2014), and the City of Split (N = 512; recruited in 2012-2013). The participants were recruited in the study following general practitioner’s advice, newspaper and radio announcements, or distribution of posters and leaflets. In order to participate, the participants had to be of age (18 or more years) and had to sign the informed consent prior to the enrolment. The study protocol was approved by the ethical board of the Medical School, University of Split (approval number 2181-198-03-04/10-11-0008).

Every participant provided a blood and urine sample following an overnight fast and gave the medical history, after which they filled in an extensive self-administered questionnaire, consisting of questions on dietary habits, smoking, alcohol consumption, physical activity, and socioeconomic status. Additionally, anthropometric and clinically relevant measurements were performed by trained medical doctors and nurses using standard operating procedures.

### Mediterranean diet assessment

A food frequency questionnaire was used to assess the dietary pattern. It consisted of 55 questions, with 6 available answers regarding the frequency of consumption (every day, 2-3 times a week, once a week, once a month, rarely, never). These questions were about olive oil and other fat consumption, milk and dairy products, various groups of vegetables, fruit, nuts, legumes, various meats, fish, and sea foods, eggs, sweets, potatoes, rice, pasta, and bread. Additionally, there were 4 questions about wine (red and white) and *bevanda* (mixture of red or white wine and water) consumption expressed in liters per week. According to the proposed MDSS approach (20), we created 14 categories of foods that comprised Mediterranean diet: fruit (including 2 questions; fresh and dried fruit), vegetables (5 questions; leafy, rooted, cruciferous, tomatoes, canned, and pickled vegetables), cereals (5 questions; white bread, wholegrain bread, rice, pasta, muesli), potatoes, olive oil, nuts, dairy products (5 questions; milk, yoghurt, sour cream, hard cheese, cottage cheese), legumes, eggs, fish (4 questions; blue fish, white fish, mollusks, octopus), white meat (2 questions; chicken, turkey), red meat (6 questions; beef, calf, pork, lamb, sausages, pancetta), sweets (7 questions; cakes, chocolate, cookies, bonbons, jam, sweetened fruit juice, fizzy drinks), fermented beverages (4 questions; red and white wine, red and white *bevanda*). If the participants reported daily consumption of olive oil, fruit, vegetables, and cereals, they were awarded 3 points, for daily consumption of nuts and dairy products they were awarded 2 points, and for the consumption of the remaining 8 categories they were awarded 1 point. These included potatoes (if consumed ≤3 servings/week), legumes (≥2 servings/week), eggs (2-4 servings/week), fish (≥2 servings/week), white meat (2 servings/week), red meat (<2 servings/week), sweets (≤2 servings/week), and fermented beverages (1 glass/d of wine or *bevanda* for women, 2 glasses/d for men; beer was not included) (20). In case these guidelines were exceeded for meat, eggs, potatoes, sweets, and wine or not reached for other categories, the participant would get 0 points. In this way, the foods that are more beneficial for health and should be consumed several times a day bring greater weight to the final score, while the foods like red meat, eggs, potatoes, and sweets that should be kept at low frequency of consumption bring lesser weight to the final score. The MDSS is based on the new Mediterranean diet pyramid, which places vegetables at the base of the pyramid, alongside with the cereals, olive oil, and fruit (28). Since our questionnaire allowed the highest frequency of consumption to be once a day, we combined 5 different types of vegetables (leafy, rooted, cruciferous, tomatoes, canned, and pickled vegetables) and thus awarded 3 points only to those participants who reported daily consumption of at least 2 types of vegetables or to those who consumed at least one type of vegetables every day plus the combination of other types, which added up to a consumption frequency of ≥7 days a week. The maximum possible MDSS score was 24 points, and the cut-off of ≥13.5 points was considered as good compliance (20). We excluded 125 participants from the analysis due to missing values needed for MDSS items calculation.

### Lifestyle and socioeconomic characteristics

Besides diet, we assessed other lifestyle indicators, such as smoking and physical activity. According to the smoking status, we divided participants into smokers (those who reported current smoking or ceased smoking less than a year ago) and non-smokers. Physical activity was assessed during the working part of the day and during the leisure time, with four responses: intensive, moderate, light, and sitting. The number of completed years of schooling (educational attainment) was used in the estimation of the socioeconomic status.

### Statistical analysis

Categorical variables are shown as numbers and percentages, and numerical variables are shown as median and interquartile ranges (IQR), due to non-normal distribution assessed using Kolmogorov-Smirnov test. Χ^2^ test was used to investigate the differences between groups for categorical variables, and Kruskal-Wallis test was used for numerical variables. Mann-Whitney U test was used as a *post-hoc* test for numerical variables and Χ^2^ test for categorical variables. Correlation between MDSS score and age and education expressed as years of schooling was performed using Spearman rank test. Finally, multivariate logistic regression analysis was used to investigate the characteristics associated with greater compliance with the Mediterranean diet, using the upper quartile of 14 points as a cut-off, which also corresponds to a proposed MDSS cut-off for good compliance with the Mediterranean diet (20). The model included six covariates: age, sex, place of residence, years of schooling, smoking, and physical activity. Age and years of schooling were classified into categories in order to provide better understanding of the results. Age was classified into three categories (18-34.9 years, 35-64.9, and ≥65 years), while years of schooling were divided in four categories (<8 years, 8-10, 11-12, and ≥13 years). In all instances we provided odds ratios (OR) and 95% confidence intervals (CI). Significance level was set at *P* < 0.05. Statistical software used was IBM SPSS Statistics v19 (IBM, Armonk, NY, USA).

## Results

The analysis included 2768 participants from three study sites (Table 1). The overall median MDSS score was 11 out of 24 points (IQR 8-13). The highest median MDSS score of 12 points was recorded on Vis (IQR 9-14), followed by 11 points in Split (IQR 8-15) and 10 points on Korčula (IQR 8-13). There was a significant difference between participants from Korčula and Vis as well as between Korčula and Split (both *P* < 0.001), while participants from Vis did not differ from those from Split (Table 1). This difference was observed across all three age groups, with the exception for the youngest age group, where participants from Korčula did not differ from participants from Vis (Table 1). There was a wide range of variability in the compliance with the MDSS components, from only 3% of participants meeting the requirements for nuts consumption on Vis, to as much as 95% for cereals (Table 1). Overall, low percentages of participants met some of the MDSS components criteria; only 28% of participants from Korčula adhered to the daily vegetable consumption requirement, 33% from Vis, and 42% from Split. MDSS recommendations for dairy products, legumes, eggs, and wine consumption were met by 17%-26% of the participants, while those for meat and sweets were met by 29%-40% of the participants (Table 1). In total, 635 (22.9%) participants reported a dietary pattern adherent to the Mediterranean diet pattern according to the MDSS criteria. Participants from Vis displayed less difference from participants from Split than from participants from Korčula regarding the adherence to the MDSS components (Table 1). The prevalence of compliance to the guidelines for sweets, white meat, eggs, legumes, and dairy products did not exhibit difference across three subgroups according to the place of residence (Table 1).

**Table 1:** Participants’ demographic characteristics and prevalence of compliance with 14 Mediterranean Diet Serving Score (MDSS) components and overall good Mediterranean diet adherence (MDSS≥14 points) according to the place of residence

	Korčula island N = 1874	Vis island N = 385	Split N = 509	Overall *P* (post-hoc test *P* values)
Sex; n (%)				0.301 (0.238*;0.224†; 0.939^‡^)
men	685 (36.6)	153 (39.7)	201 (39.5)	
women	1189 (63.4)	232 (60.3)	308 (60.5)	
Age (years); median (interquartile range, IQR)	55.0 (40.7-65.3)	63.5 (54.1-73.1)	58.0 (47.0-66.0)	<0.001(<0.001*; <0.001†; <0.001^‡^)
Years of schooling; median (IQR)	12 (9-12)	11 (6-12)	12 (12-16)	<0.001 (<0.001*; <0.001†; <0.001^‡^)
Smoking; n (%)				<0.001 (0.001*; <0.001†; <0.001^‡^)
current smokers	522 (27.9)	99 (25.7)	84 (16.5)	
ex-smokers	369 (19.7)	49 (12.7)	132 (25.9)	
never-smokers	983 (52.4)	237 (61.6)	293 (57.6)	
Physical activity; n (%)				<0.001 (<0.001*; <0.001†; <0.001^‡^)
light	341 (18.2)	148 (38.4)	136 (26.7)	
moderate	1210 (64.6)	200 (51.9)	356 (69.9)	
intensive	174 (9.3)	21 (5.5)	8 (1.6)	
Body mass index (kg/m^2^); median (IQR)	26.8 (24.0-29.7)	28.0 (25.6-30.9)	27.2 (24.6-30.0)	<0.001 (<0.001*;0.011†; 0.003^‡^)
MDSS; median (IQR)	10 (8-13)	12 (9-14)	11 (8-15)	<0.001 (<0.001*; <0.001†; 0.401^‡^)
MDSS in 18-34.9 age group; median (IQR)	9.0 (7.0-11.0)	11.5 (6.8-13.3)	10.0 (8.0-14.0)	0.006 (0.242*;0.002†; 0.969^‡^)
MDSS in 35-64.9 age group; median (IQR)	10.0 (8.0-13.0)	11.0 (9.0-13.0)	11.0 (8.0-14.0)	<0.001 (0.012*; <0.001†; 0.397^‡^)
MDSS in ≥65.0 age group; median (IQR)	11.0 (9.0-14.0)	13.0 (10.0-15.0)	13.0 (10.0-15.0)	<0.001 (<0.001*;0.035†; 0.377^‡^)
MDSS components; n (%)				
fruit	937 (50.0)	228 (59.2)	320 (62.9)	<0.001 (0.001*; <0.001†; 0.268^‡^)
vegetables	521 (27.8)	127 (33.0)	216 (42.4)	<0.001 (0.041*; <0.001†; 0.004^‡^)
cereals	1625 (86.7)	365 (94.8)	405 (79.6)	<0.001 (<0.001*; <0.001†; <0.001^‡^)
potatoes	1229 (65.6)	334 (86.8)	462 (90.8)	<0.001 (<0.001*; <0.001†; 0.066^‡^)
olive oil	1283 (68.5)	296 (76.9)	328 (64.4)	<0.001 (0.001*; 0.085†; <0.001^‡^)
nuts	86 (4.6)	10 (2.6)	57 (11.2)	<0.001 (0.078*; <0.001†; <0.001^‡^)
dairy products	339 (18.1)	84 (21.8)	106 (20.8)	0.132 (0.088*; 0.160†; 0.742^‡^)
legumes	412 (22.2)	78 (20.3)	122 (24.0)	0.406 (0.454*; 0.341†; 0.196^‡^)
eggs	462 (24.7)	80 (20.8)	134 (26.3)	0.148 (0.105*; 0.440†; 0.058^‡^)
fish	1161 (62.0)	252 (65.5)	286 (56.2)	0.013 (0.196*; 0.018†; 0.006^‡^)
white meat	758 (40.4)	136 (35.3)	185 (36.3)	0.069 (0.067*; 0.093†; 0.778^‡^)
red meat	548 (29.2)	127 (33.0)	201 (39.5)	<0.001 (0.144*; <0.001†; 0.050^‡^)
sweets	597 (31.9)	105 (27.3)	164 (32.2)	0.185 (0.077*; 0.876†; 0.122^‡^)
wine	315 (16.8)	125 (32.5)	136 (26.7)	<0.001 (<0.001*; <0.001†; 0.064^‡^)
MDSS≥14 points; n (%)	352 (18.8)	123 (31.9)	160 (34.4)	<0.001 (<0.001*; <0.001†; 0.885^‡^)

*Post-hoc test *P* values: Korčula vs Vis.

†Post-hoc test *P* values: Korčula vs Split.

‡Post-hoc test *P* values: Vis vs Split.

**Figure 1:**
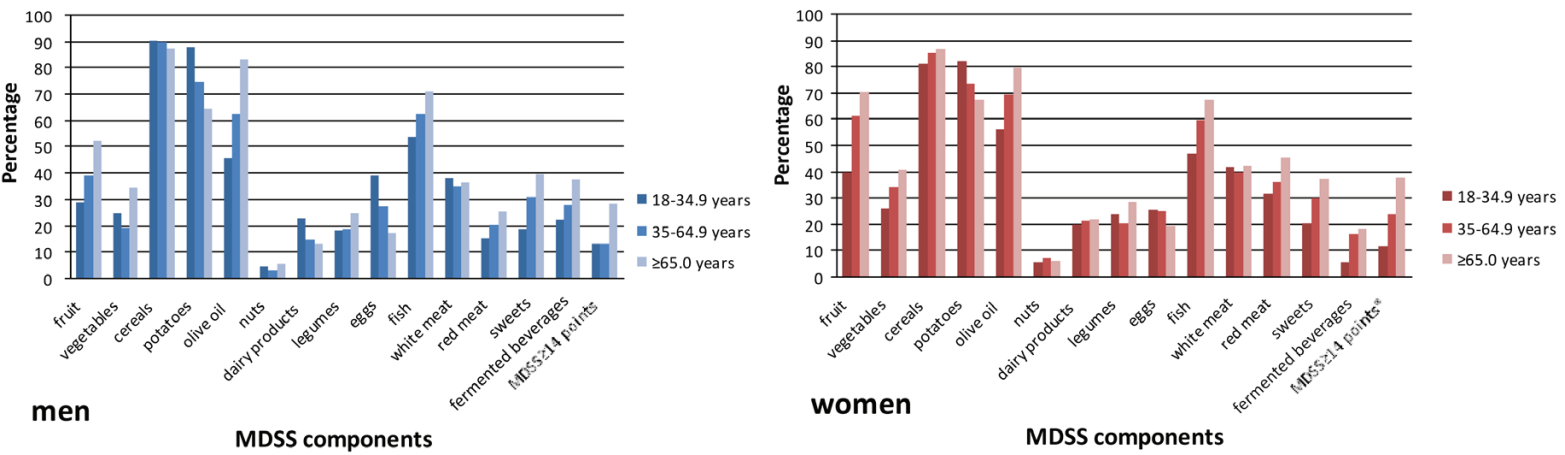
Prevalence of compliance with 14 Mediterranean Diet Serving Score (MDSS) components and overall good Mediterranean diet adherence (MDSS≥14 points), according to sex and age groups (significant differences at the level of *P* < 0.05 between age groups are denoted with asterisk; χ^2^ test).

Breakdown by sex and age suggested somewhat better indices in women and older age group, with only one significant result: good compliance to the Mediterranean diet differed according to age groups only in women (*P* = 0.024) (Figure 1).

Breakdown into three groups according to the participant’s age revealed higher MDSS scores in the elderly group, with a significant difference between all three age groups (all *P* < 0.001) (Table 2). Correlation between age and MDSS score was also significant in the whole sample (ρ = 0.256, *P* < 0.001; data not shown), as well as in the subgroups according to the place of residence (ρ = 0.243, *P* < 0.001 in Korčula; ρ = 0.276, *P* < 0.001 in Vis; ρ = 0.189, *P* < 0.001 in Split). Education (years of schooling) was not correlated with the MDSS score (ρ = -0.006, P = 0.736 in the whole sample; ρ = -0.045, *P* = 0.057 in Korčula; ρ = -0.045, *P* = 0.387 in Vis; ρ = 0.113, *P* = 0.011 in Split).

**Table 2:** Participants’ demographic characteristics and prevalence of compliance with 14 Mediterranean Diet Serving Score (MDSS) components and overall good Mediterranean diet adherence (MDSS≥14 points), according to the age group

	Age groups			Overall *P* (post-hoc test *P* values)
	18-34.9 years (N = 372)	35-64.9 years (N = 1617)	65 and more (N = 779)	
Sex; n (%)				0.016 (0.086*;0.772†; 0.007^‡^)
men	149 (40.1)	571 (35.3)	319 (40.9)	
women	223 (59.9)	1046 (64.7)	460 (59.1)	
Years of schooling; median (interquartile range, IQR)	12.0 (11.0-15.0)	12.0 (11.0-13.0)	11.0 (6.0-12.0)	<0.001 (<0.001*; <0.001†; <0.001^‡^)
Smoking; n (%)				<0.001 (<0.001*; <0.001†; <0.001^‡^)
current smokers	163 (45.4)	478 (32.4)	64 (9.2)	
never-smokers and ex-smokers	209 (54.6)	1139 (67.6)	715 (90.8)	
Physical activity; n (%)				<0.001 (0.130*; <0.001†; <0.001^‡^)
light	85 (23.3)	284 (18.7)	256 (36.1)	
moderate	252 (69.0)	1102 (72.5)	412 (58.0)	
intensive	28 (7.7)	133 (8.8)	42 (5.9)	
MDSS; median (IQR)	9.0 (7.0-11.0)	11.0 (8.0-13.0)	12.0 (9.0-14.0)	<0.001 (<0.001*; <0.001†; <0.001^‡^)
MDSS components; n (%)				
fruit consumption	132 (35.5)	863 (53.4)	490 (62.9)	<0.001 (<0.001*; <0.001†; <0.001^‡^)
vegetables	95 (25.5)	471 (29.1)	298 (38.3)	<0.001 (0.166*; <0.001†; <0.001^‡^)
cereals	316 (84.9)	1403 (86.8)	676 (86.8)	0.632 (0.356*; 0.400†; 0.993^‡^)
potatoes	314 (84.4)	1195 (73.9)	516 (66.2)	<0.001 (<0.001*; <0.001†; <0.001^‡^)
olive oil	193 (51.9)	1082 (66.9)	632 (81.1)	<0.001 (<0.001*; <0.001†; <0.001^‡^)
nuts	19 (5.1)	88 (5.4)	46 (5.9)	0.835 (0.796*; 0.584†; 0.644^‡^)
dairy products	78 (21.0)	308 (19.0)	143 (18.4)	0.571 (0.398*; 0.293†; 0.685^‡^)
legumes	80 (21.5)	320 (19.8)	212 (27.2)	<0.001 (0.473*; 0.037†; <0.001^‡^)
eggs	115 (30.9)	416 (25.7)	145 (18.6)	<0.001(0.041*; <0.001†; <0.001^‡^)
fish	185 (49.7)	978 (60.5)	536 (68.8)	<0.001 (<0.001*; <0.001†; <0.001^‡^)
white meat	150 (40.3)	618 (38.2)	311 (39.9)	0.617 (0.452*; 0.897†; 0.423^‡^)
red meat	93 (25.0)	493 (30.5)	290 (37.2)	<0.001 (0.037*; <0.001†; 0.001^‡^)
sweets	74 (19.9)	493 (30.5)	299 (38.4)	<0.001 (<0.001*; <0.001†; <0.001^‡^)
wine	45 (12.1)	327 (20.2)	204 (26.2)	<0.001 (<0.001*; <0.001†; 0.001^‡^)
MDSS≥14 points; n (%)	46 (12.4)	324 (20.0)	265 (34.0)	<0.001 (0.001*; <0.001†; <0.001^‡^)

*Post-hoc test *P* values: 18-34.9 years vs 35–64.9 years.

†Post-hoc test *P* values: 18-35 years vs ≥65 years.

‡Post-hoc test *P* values: 34.9-64.9 years vs ≥65 years.

Only 12% of participants from the youngest group met the MDSS cut-off criterion, while the corresponding figure in the oldest age group was 34% (Table 2). White meat, dairy products, nuts, and cereals were the only MDSS components that did not exhibit difference in compliance across three age groups (Table 2).

Logistic regression analysis revealed several variables to be strongly associated with the good adherence to the Mediterranean diet (MDSS≥14 points). For instance, men had lesser odds of showing good compliance compared to women (OR 0.52, 95% CI 0.42-0.65, *P* < 0.001), while the youngest age group had 70% lesser odds compared to the oldest participants (OR 0.30, 95% CI 0.20-0.45, *P* < 0.001) (Table 3). Both the participants from Vis and those from Split showed greater odds for good adherence to the Mediterranean diet (OR 1.99, 95% CI 1.50-2.64, *P* < 0.001 and OR 1.67, 95% CI 1.28-2.19, *P* < 0.001, respectively) (Table 3). Physical activity, lower education, and smoking were marginal predictors or lacked significance (Table 3). The regression model yielded good data fit (Hosmer and Lemeshow *P* = 0.225, Nagelke R^2^ 0.101).

**Table 3:** Characteristics associated with good adherence to the Mediterranean diet (MDSS≥14 points) using the multivariate logistic regression analysis*

	Unadjusted odds ratio (95% confidence interval); *P*	Adjusted odds ratio (95% confidence interval); *P*
Sex		
women (Ref.)	1.00	1.00
men	0.62 (0.51-0.75); <0.001	0.52 (0.42-0.65); <0.001
Age		
65 and more (Ref.)	1.00	1.00
35-65	0.49 (0.40-0.59); <0.001	0.44 (0.35-0.56); <0.001
18-35	0.27 (0.19-0.39); <0.001	0.30 (0.20-0.45); <0.001
Place of residence		
Korčula	1.00	1.00
Split	1.98 (1.59-2.47); <0.001	1.67 (1.28-2.19); <0.001
Vis	2.03 (1.59-2.59); <0.001	1.99 (1.50-2.64); <0.001
Years of schooling		
13 or more	1.00	1.00
under 8	1.04 (0.77-1.40); 0.788	0.57 (0.39-0.83); 0.003
8-10	0.80 (0.59-1.08); 0.140	0.70 (0.49-0.99); 0.045
11-12	0.56 (0.45-0.69); <0.001	0.61 (0.48-0.79); <0.001
Smoking		
non-smokers and ex-smokers	1.00	1.00
smokers	0.61 (0.49-0.77); <0.001	0.77 (0.60-0.99); 0.045
Physical activity		
intensive	1.00	1.00
light	1.17 (0.80-1.70); 0.420	0.61 (0.40-0.93); 0.021
moderate	0.98 (0.69-1.40); 0.934	0.71 (0.48-1.04); 0.080

*MDSS – Mediterranean Diet Serving Score.

## Discussion

This study revealed rather unsatisfactory Mediterranean diet consumption in southern Croatia. The median MDSS score was as low as 10 out of 24 points among participants from Korčula, 11 among those from Split, and 12 among those from Vis. A similar study performed in Spain reported an average MDSS score among women of 12.5 ± 2.7 (20). The percentage of participants who adhered to the Mediterranean diet in our study was comparable to or lower than figures obtained in other Mediterranean countries, eg, 32% in Greece (29) and 18% in Italy (30). However, it is not possible to make any in-depth comparisons between these studies and our results due to methodological differences and the differences in the definition of the Mediterranean diet adherence (17). However, the majority of the studies do report a rather low percentage of people whose dietary pattern resembles the Mediterranean diet, especially in the youngest generations (30-33).

The required high intake of vegetables (≥2 servings/main meal) was poorly met in this study, especially among participants from Korčula (28%), younger people (26%), and men (as low as 19% in the middle age group). Although rather low, these percentages surpass those from Spain, where only 11% of women met the same criterion (20). This suggests that this is a very restrictive criterion, although it is one of the most important ones according to the recent scientific evidence on the health benefits of the plant-based diet compared to the meat-based diets (34,35).

Fruit consumption showed overall better results, with the lowest consumption among the youngest men (29%) and the highest among the oldest women (70%). Olive oil intake was commonly reported, but slightly less commonly than in Spain (20). Satisfactory fish consumption was generally present in more than 50% of the participants, with the highest proportion recorded among elderly men (71%) and the lowest among youngest women (47%).

The worst result for a particular component was recorded for nuts consumption, where as little as 3% of people from Vis reported eating nuts every day, and the situation was not much better among the participants from Korčula (5%) and Split (11%). This is particularly unfortunate because a recent field trial showed that daily consumption of mixed nuts (30 g/d) reduced the incidence of major cardiovascular events by 28% in people at high cardiovascular risk, but with no cardiovascular disease and after a median follow-up of 4.8 years (16). Also, an observational study showed a 47% reduction in all-cause mortality and 36% reduction in cancer deaths in people who ate nuts ≥8 times/month, compared to those who never ate nuts during a median follow-up of 4.3 years (36). Additionally, consumption of nuts was also shown to be protective against cognitive decline in elderly people (37), or even to improve cognitive function (7).

A better compliance with the overall Mediterranean diet, as well as with the majority of the MDSS components, was recorded among the oldest participants, especially women, even though without reaching statistical significance, which is in line with many previous studies (20,38,39). Participants from Vis had the highest prevalence of compliance to MDSS components and overall the highest prevalence of Mediterranean diet adherence (MDSS≥14 points). After stratification according to age groups, participants from Korčula showed the poorest compliance, while participants from Vis and Split showed a similar dietary pattern.

Earlier studies from Croatian islands also revealed a worrisome pattern of dietary habits, with a predominant shift toward higher consumption of meat, pasta, and cakes (23). A study from the coastal region of Adriatic Sea yielded even more devastating results; only 2.4% of the general population in urban areas and 3.4% in rural areas ate Mediterranean diet defined as “daily intake of fruits and vegetables, brown bread and whole grains, using olive oil as the main source of fat, consumption of fish and moderate wine drinking with meals” (40). This departure from the traditional diet, as well as other lifestyle factors, such as high prevalence of smoking in both island populations and overall lower levels of physical activity, as demonstrated in this study, might as well be responsible for the observed high burden of overweight and hypertension (41), hyperlipidemia (24), type 2 diabetes (42), and metabolic syndrome (43) in the Mediterranean region of Croatia. This detrimental situation is also reflected in the concurrent mortality patterns (unpublished data, Rehberg J et al, 2016).

Limitations of this study include the use of the convenient sampling approach, possible recall bias, and a slightly different food frequency questionnaire from the one used in the study that proposed the MDSS score (20). A number of other possible confounders were omitted, but we applied a validated and simple tool to assess the compliance with the Mediterranean diet, which enables wider-scale international comparisons. One of the confounding factors not taken into account is the morbidity pattern, especially age-related diseases, which could have affected dietary habits as well as the adherence to the Mediterranean diet. Given the high prevalence of chronic diseases in the investigated population (41-43), it would be expected that people, especially elderly persons, would show greater adherence to the healthy Mediterranean diet as a form of both preventive measure and treatment, but in the oldest group we demonstrated the prevalence of good Mediterranean diet adherence of only 34%.

This study suggests a diminishing adherence to the traditional Mediterranean diet and lifestyle, especially in the younger generations, which needs to be considered as a public health priority and reversed not only for the population health benefits, but also as a part of the cultural heritage safe-keeping. In addition, adherence to the Mediterranean diet has much more favorable environmental footprint (44), which is of utmost importance in small and fragile habitats, such as the remote islands.
